# Juvenile Clinically Amyopathic Dermatomyositis: A Case Report and Review of Literature

**Published:** 2019

**Authors:** Vadood JAVADI PARVANEH, Mehrdad YASAEI, Khosro RAHMANI, Yalda NILIPOUR

**Affiliations:** 1 Department of Rheumatology, Mofid Children’s Hospital, Shahid Beheshti University of Medical Sciences, Tehran, Iran; 2 Pediatric Pathology Research Center, Mofid Children’s Hospital, Shahid Beheshti University of Medical Sciences, Tehran, Iran

**Keywords:** Juvenile dermatomyositis, Juvenile clinically amyopathic dermatomyositis, Children, pediatrics, Dermatomyositis sine myositis

## Abstract

Juvenile clinically amyopathic dermatomyositis (juvenile CADM) is a rare rheumatologic disease in children defined as the presence of the hallmark cutaneous features of dermatomyositis in absence of muscle involvement**. **In this article, we report an Iranian 14.5-year-old girl presented to Rheumatology Clinic of Mofid Children's Hospital, Tehran, Iran in Jan 2016 with cutaneous complaints diagnosed with juvenile CADM. Finally, we provide a literature review of previous studies on juvenile CADM.

## Introduction

Juvenile clinically amyopathic dermatomyositis (juvenile CADM) or dermatomyositis sine myositis is a rare rheumatic disease in children which the child initially presents with the classic rash of dermatomyositis in the absence of clinically muscle involvement (1, 2). 

Herein we present a teenage girl with juvenile CADM from Tehran, Iran.

## Case Report

A 14.5-year-old girl was referred to the Rheumatology Clinic, Mofid Children's Hospital, Tehran, Iran in Jan 2016 with a history of weight loss, photosensitivity and violaceous rashes on her upper eyelids about 6 months ago. She had no complaints of fever, weakness, malaise, myalgia or arthralgia. The informed consent was obtained from the parents of the girl.

She was first treated by a dermatologist with a course of topical betamethasone, intramuscular glucocorticoids, and chlorpheniramine, but there was no significant improvement. On physical examination, there were bilateral heliotrope rashes on her upper eyelids extended to the ears. Malar rashes on her cheeks were noted, connecting together on the nose. Erythematous papular lesions were seen on her anterior and posterior chest (V sign), anterior and posterior neck (shawl sign) and also on her abdomen and flanks. She showed violaceous papules on her interphalangeal joints (Gottron’s papules) and extensor aspect of her forearms. Nail folds changes with periungual erythema were prominent. On her palate erythema and petechia were revealed. Proximal and distal muscle strength of limbs were normal and no muscle tenderness was noted.

The laboratory tests showed a complete blood count with a microchrome microcytic anemia compatible with minor beta thalassemia. Blood Urea Nitrogen (BUN), creatinine (Cr), creatine phosphokinase (CPK), aldolase, lactate dehydrogenase (LDH), aspartate transaminase (AST), alanine transaminase (ALT), C3, C4, CH50, protein C and S were all within normal range. Fluorescent antinuclear antibodies(FANA), anti-cyclic citrullinated peptide (CCP), perinuclear- antineutrophil cytoplasmic antibodies (P-ANCA), cytoplasmic-antineutrophil cytoplasmic antibodies (C-ANCA), anti-double strand-DNA (ds-DNA), anti topoisomerase1 (SCL-70), anti jo1, anticentromere and anti-ribonucleoprotein (RNP) were negative.

**Figure 1:**
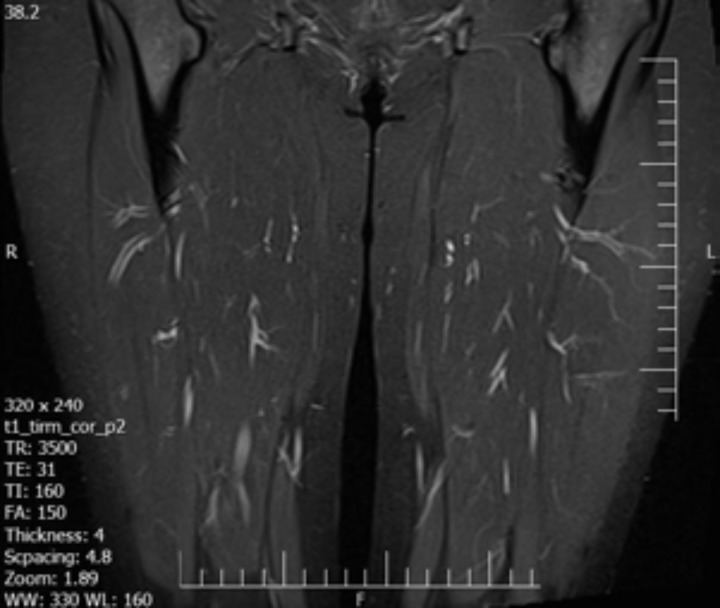
Muscle MRI showed no abnormal signal

**Figure 2:**
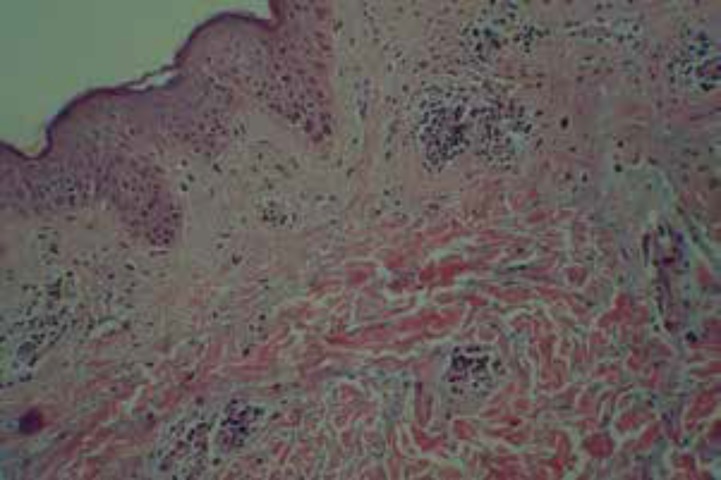
Skin biopsy showed mild lichenoid reaction and perivascular chronic inflammatory cell infiltration of dermal layer and some epidermotropism and no spongiosis

**Figure 3:**
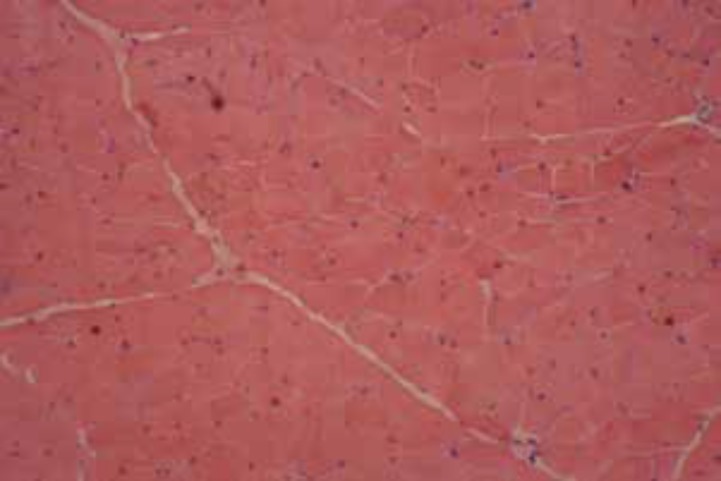
Muscle biopsy showed slight fiber variation and no necrosis/regeneration and perifascicular atrophy

Capillaroscopy of nail fold showed angiogenesis, avascular area, irregularly enlarged loops, isolated micro-bleeding, megcapillary, and tortuosity. Electromyography (EMG) and nerve conduction velocity (NCV) were normal. In abdominopelvic sonography, liver echogenicity was mildly increased suggesting of grade I fatty liver. MRI of the thigh was normal (Figure 1). Skin biopsy of the trunk lesions reported as lichenoid tissue reaction with superficial perivascular dermatitis and dermal mucin deposition compatible with collagen vascular disease (Figure 2). Muscle biopsy was taken from biceps muscle and neither inflammation nor perifasicular atrophy pattern was seen (Figure3). Cardiopulmonary investigation showed no abnormality on chest X-ray and echocardiography.

Treatment was started on Feb 2016 with ophthalmic ointment of hydrocortisone, hydroxychloroquine sulfate 6.5 mg/kg/d and prednisolone 1 mg/kg/d.

After 10 wk, all cutaneous findings were resolved; therefore, the dosage of prednisolone was gradually tapered to 0.25 mg/kg/d over 6 months. During 36 month’s follow-up (Feb 2019), the patient had no manifestations of muscular, cutaneous and cardiopulmonary involvement.

## Discussion

Clinically amyopathic dermatomyositis (CADM) is defined as biopsy-confirmed hallmarked cutaneous manifestations of dermatomyositis without muscle weakness and with normal serum muscle enzymes. If more diagnostic muscle tests are performed, the results will be normal (1, 2).

About 5%-20% of dermatomyositis patients are estimated to represent CADM (3-5). Childhood presentation of CADM is rare and there are not many studies on the epidemiology of juvenile CADM (2, 6).

From the first cases described in 1963 (7), several cases of CADM have been reported describing the clinical feature, laboratory data, treatment courses, and disease complications. Some of these cases were patients younger than 18 yr of old classified as juvenile CADM. A systematic review for juvenile CADM was published and showed that in contrast with the adult-onset CADM, morbidity, and mortality in juvenile cases were related to calcinosis and severe vasculopathy, and no associated interstitial lung disease (ILD) was reported (2, 8).

Although lung disease is rare among juvenile patients, there are some reports since 2007 about ILD in children with CADM (9-17). Four cases were reported of ILD among juvenile CADM, 2 with rapidly progressive ILD (RP-ILD) who died from diffuse alveolar damages and 2 with a chronic course (17). An 11-yr-old patient was reported with CADM and ILD (11). An 18-yr-old male and 16 yr old female reported with having RP-ILD (12). An 18-yr-old female had radiographic changes indicative of lung fibrosis (13). Eleven juvenile DM patients were described with ILD (5 with classic DM and 6 with CADM), 6 of them had RP-ILD and only 2 of them survived through the treatment (9). A 16-yr-old boy with hepatosplenomegaly and rapidly progressive interstitial pneumonia was reported; the patient died after 1 year (14). Juvenile CADM initiating with acquired inflammatory Blaschko-linear dermatosis in a 21-yr-old woman who had the skin lesions from 6-year-ago. The patient has signs of ILD in high resolution computerized tomography (HRCT) and pulmonary function test (PFT) (15). A case of hyper-ferritinemia syndrome evolving was reported in recurrent macrophage activation syndrome, as onset of amyopathic juvenile dermatomyositis. The patient was a 13-yr-old girl diagnosed for ILD due to cardiopulmonary insufficiency (16).

ILD, especially RP-ILD, occurs more in CADM patients than classic DM and has a poorer prognosis (10, 18-20), several studies have been done to find the association between specific autoantibodies and the rate of ILD in CADM patients. Autoantibodies against 140-kDa cytoplasmic protein (Anti-CADM 140) was first described in adults with CADM in the Japanese population (21). The antigen of this antibody is an RNA helicase encoded by melanoma differentiation-associated gene 5 antibodies (MDA5) which is an important molecule in innate immunity (22, 23). Later a strong association was seen between the RP-ILD and the presence of anti-CADM 140 (anti-MDA-5) in juvenile patients with both classic DM and CADM (9, 10). Moreover, anti-MDA-5 antibodies are useful in predicting and evaluating response to treatment (18). Better response to treatment and survival was seen in patients who had a lower mean titer of anti-MDA antibody prior to the start of treatment (24).

Anti-MDA-5 is positive in juvenile chronic ILD cases as it is in rapidly progressive ones. Anti-MDA5 was positive in classic JDM with RP-ILD (17). In a study, including 2 juvenile patients, HLA– DRB1*0101/*0405 is associated with susceptibility to anti-MDA5 positive patients with CADM (12). Other autoantibodies also have been shown in CADM patients including Transcriptional Intermediary Factor 1-gamma (TIF1-gamma) and anti-aminoacyl-tRNA synthetase antibodies (anti-Jo-1, anti-EJ, anti-PL-7, anti-PL-12, and anti-KS) (11, 25) but their presence in juvenile patients has not been identified.

There are no randomized control clinical trials on management of juvenile CADM. In a systematic review, topical corticosteroids were the most common treatment modality; however, many of the studies have not mentioned the management of the disease (All references in 2). In adult CADM cases, topical treatment (including sun protection, topical corticosteroids, topical calcineurin inhibitors) and systemic treatment (including oral corticosteroids, dapsone, antimalarials, IVIG, methoterexate, azatioprine, mycophenolate mofetil) have been administered in different studies (26).Using systemic corticosteroids in juvenile CADM has remained controversial. As an increased incidence of calcinosis was noted in patients with cutaneous findings preceding muscle symptoms (27). Early initial systemic management by corticosteroids may decrease the incidence of calcinosis in classic DM patients (28-30). On the other hand, there was no significant association between systemic treatment and the incidence of calcinosis in classic DM or juvenile CADM (31-33), plus the rate of calcinosis in juvenile CADM is very low in contrast to classic DM (2).

Hydroxychloroquine showed to have successful outcomes in resolving the cutaneous lesions of classic DM both in adults and juvenile patients (34, 35). There are few case reports about the use of hydroxychloroquine in juvenile CADM patients (16, 36-40).

Methotrexate in combination with corticosteroids showed satisfying results in juvenile CADM patients (32, 41, 42). The case of reference 6 treated with prednisolone and methotrexate, is on 2- year remission with 5-yr follow-up.

Recently, a treatment strategy for juvenile CADM patients was suggested but there are no recommendations in patients complicating with ILD (43).

Intensive therapy was administered in patients with ILD. Methylprednisolone pulse therapy, high dose oral prednisolone, cyclosporine A, cyclophosphamide, mizoribine and methotrexate reported in different studies (Sato Kobayashi abe poddighe cao) (9, 10, 13, 14, 16).

**In conclusion, **Juvenile CADM is a rare condition among children. Although muscle weakness is absent in these patients and the rate of calcinosis is lower in contrast with classic JDM patients, they are at higher risk for ILD. Presence of anti MDA5 antibody has been shown to have a strong association with ILD in CADM patients and can be used as a prognostic factor as well as evaluating response to treatment. There is still a big controversy in managing juvenile CADM patients. The management of CADM is mostly based on treatment protocols used in classic DM patients and there are no specific clinical trials in juvenile CADM cases. However, in case of ILD intensive therapy seems to be the only option for reducing the mortality rate.
